# Preparation, Properties and Application Research of PVA/ANF/NaCl Composite Organic Hydrogel

**DOI:** 10.3390/gels12050442

**Published:** 2026-05-19

**Authors:** Guofan Zeng, Jiaqi Zhu, Zehong Wu, Yihan Qiu, Mingcen Weng

**Affiliations:** 1 Department of Physical Education, Fujian University of Technology, Fuzhou 350118, China; 2 Institute of Biology and Chemistry, Fujian University of Technology, Fuzhou 350118, China

**Keywords:** PVA/ANF/NaCl composite organohydrogel, aramid nanofibers, flexible sensor, strain sensing, pressure sensing, triboelectric nanogenerator, wearable electronics

## Abstract

Polyvinyl alcohol (PVA)-based hydrogels suffer from insufficient mechanical strength, while aramid nanofibers (ANF) have intrinsic insulation that limits their sensing applications, and the synergistic effect of composite fillers remains underexplored. This study aims to develop a multifunctional PVA/ANF/NaCl composite organohydrogel for high-performance flexible sensors. The gel was fabricated via freeze–thaw crosslinking, solvent exchange and NaCl impregnation, with systematic investigations of its microstructure, mechanical, electrical and multifunctional sensing properties, and a corresponding triboelectric nanogenerator (TENG) and self-powered handwriting recognition system were constructed. Results show that 2% ANF significantly enhances the gel’s mechanical performance, 0.5 M NaCl achieves optimal mechanical-electrical balance, the gel-based sensor exhibits excellent distance, pressure and strain sensing with high cyclic stability, the TENG delivers stable electrical output, and the recognition system achieves 95% accuracy on the test set. This work provides a new material and design strategy for advanced flexible electronic devices.

## 1. Introduction

In recent years, nanofibers have been widely used as mechanically reinforcing fillers to construct gel network structures [1]. Among them, aramid nanofibers (ANFs), which feature high strength, low density, and excellent thermal stability, can significantly improve the mechanical properties of polyvinyl alcohol (PVA)-based conductive gels [2,3,4]. However, the intrinsic insulating nature of ANFs makes it difficult to meet the conductivity requirements of sensors when used alone. Therefore, this study innovatively introduces NaCl as an electrolyte to compensate for the electrical deficiency of ANFs through an ionic conductive mechanism, thereby constructing a PVA/ANF/NaCl composite organohydrogel system, in which the Na^+^ and Cl^−^ ions generated by the dissociation of NaCl form continuous ion channels in the gel network and further enhance the structural stability of the gel network via ion–hydrogen bond synergistic interactions [5,6].

Although numerous studies have reported flexible sensors based on PVA composite organohydrogels modified with single fillers, the synergistic effects of composite fillers have not been fully explored [7,8,9]. Most existing PVA-based conductive hydrogel sensors still face the inherent trade-off between mechanical robustness and ionic conductivity: enhancing mechanical strength often requires introducing insulating fillers that reduce electrical conductivity, while improving conductivity by increasing electrolyte content usually leads to deterioration of mechanical properties and structural stability. Moreover, the majority of reported hydrogel sensors only support single-mode sensing (e.g., strain or pressure), which cannot meet the growing demand for multifunctional integrated wearable electronics that can simultaneously detect multiple physical signals in complex environments [10]. In particular, the lack of systematic investigation into the synergistic interaction mechanisms between multiple functional fillers has become a major bottleneck restricting the performance optimization of composite hydrogel sensors.

Notably, flexible wearable sensors based on high-performance conductive organohydrogels have shown unprecedented potential in clinical healthcare applications beyond general consumer electronics. Longitudinal clinical monitoring requires continuous, non-invasive, and long-term tracking of patient physiological signals, which is critical for early disease diagnosis, treatment efficacy evaluation, and personalized medicine development. Traditional clinical monitoring methods are often limited to hospital settings and cannot provide real-time, continuous health data, while hydrogel-based wearable sensors with excellent biocompatibility, mechanical compliance, and multifunctional sensing capabilities can seamlessly integrate with human skin to collect high-quality physiological signals over extended periods. Recent studies have demonstrated that integrating wearable sensing technology with longitudinal clinical profiling enables holistic assessment of patient health status and early detection of treatment resistance, which can significantly improve clinical outcomes and reduce healthcare costs [11,12].

To address the above challenges, this work proposes a facile and scalable strategy to fabricate PVA/ANF/NaCl composite organohydrogels with balanced mechanical and electrical properties through freeze–thaw crosslinking, solvent exchange, and NaCl impregnation processes. We systematically investigate the effects of ANF content (0–5 wt%) and NaCl concentration (0–2 M) on the microstructure, intermolecular hydrogen bonding interactions, mechanical properties, and ionic conductivity of the composite organohydrogels, and clarify the synergistic reinforcement and conductive modulation mechanism between ANF and NaCl electrolyte. Based on the optimized composition (2 wt% ANF and 0.5 M NaCl) that achieves the best comprehensive performance, we fabricate a multifunctional flexible sensor that can simultaneously detect distance, pressure, and strain signals with high sensitivity, fast response speed, and excellent long-term cycling stability. Furthermore, we construct a vertical contact-separation mode triboelectric nanogenerator (TENG) based on this composite organohydrogel and develop a self-powered intelligent handwriting recognition system with 95% test accuracy using a multi-layer perceptron neural network, demonstrating its great application potential in mechanical energy harvesting and intelligent human–computer interaction. This work not only provides a new material platform for the development of high-performance flexible wearable electronics but also offers valuable theoretical guidance and technical support for the design of multifunctional composite hydrogels through synergistic engineering of multiple functional fillers [13,14,15,16].

## 2. Results and Discussion

### 2.1. Synthesis and Characterization of PVA/ANF/NaCl Composite Organohydrogels

#### 2.1.1. Formation Process of the PVA/ANF/NaCl Composite Organohydrogel Network

**Figure 1:**
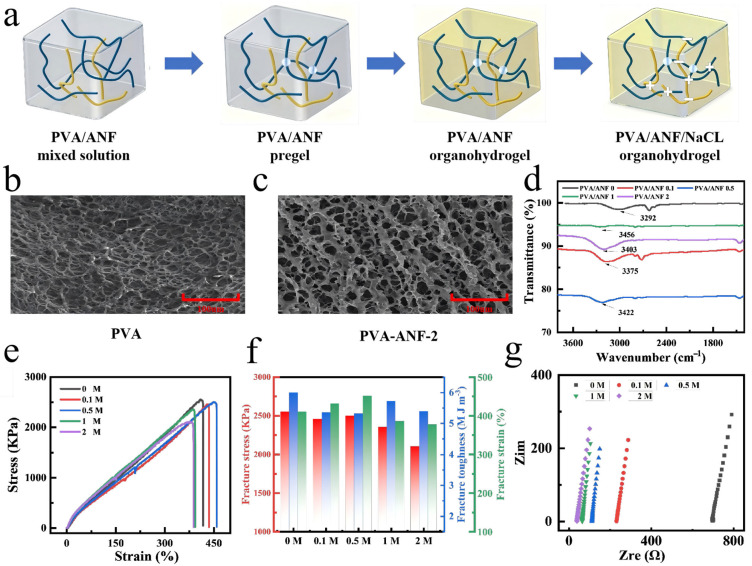
(**a**) Fabrication process of the PVA/ANF/NaCl composite organohydrogel. (**b**) Porous structure of the pure PVA gel. (**c**) Porous structure of the PVA/ANF-2% gel. (**d**) Fourier transform infrared (FTIR) spectra of PVA/ANFx organohydrogels (x = 1%, 2%, 3%, where x represents the mass fraction of ANF). (**e**) Tensile stress curves of the toughened PVA/ANF/NaCl composite organohydrogels with different NaCl contents. (**f**) Tensile stress, tensile strain and toughness of the toughened PVA/ANF/NaCl organohydrogels with different NaCl contents. (**g**) Ionic conductivity of the PVA/ANF/NaCl conductive organohydrogels at different NaCl concentrations.

The formation of the PVA/ANF/NaCl composite organohydrogel network is a multistage and complex process involving various interactions and structural evolutions, whose formation mechanism is illustrated in detail below (Figure 1a). At the initial stage, when PVA and ANF are dispersed in solution, they preliminarily construct a composite network through hydrogen-bonding interactions. The abundant hydroxyl groups on the PVA chains provide sufficient sites for hydrogen bonding, while the unique chemical structure of ANF enables tight binding with PVA chains. This interaction lays a solid “skeleton” foundation for the subsequent construction of the gel network [17,18].

Subsequently, the freeze–thaw cycling process plays a key role in further developing the gel structure. During freezing, the solvent (water) gradually forms ice crystals, and phase separation occurs between PVA and ANF, generating a PVA-rich phase and a PVA-deficient phase. As the freeze–thaw process proceeds, the formation and melting of ice crystals gradually reduce the distance between PVA chains and significantly increase the number of physical crosslinking points. These physical crosslinks act like “rivets”, which greatly enhance the mechanical stability and structural integrity of the organohydrogel, allowing the gel to withstand a certain degree of external force without deformation or fracture [19]. Meanwhile, NaCl was dissolved in a mixed solution of DMSO and water (a DMSO mass fraction of 25%). As an electrolyte, NaCl dissociates into Na^+^ and Cl^−^ ions during dissolution, creating an ionic environment in the solution. This ionic environment exerts multiple functions: on the one hand, it helps strengthen the interactions between PVA chains and promotes a more ordered arrangement; on the other hand, it significantly improves the structural stability of the solution [20]. In addition, the strong hydrogen-bonding interactions formed among NaCl, DMSO, and water during dissolution provide indispensable electrolyte support for the subsequent formation of the gel network. Next, the pre-treated PVA/ANF gel was immersed in the above NaCl–DMSO/water mixture. At this stage, the interactions between the ions in the NaCl solution and the PVA chains are further enhanced. The presence of ions strengthens the hydrogen bonding between PVA chains, similar to applying a “tensile force” that tightens the molecular chains. This tightening effect leads to a denser gel network.

#### 2.1.2. Characterization and Mechanical Properties of PVA/ANF/NaCl Composite Organohydrogels

The SEM image of pure PVA in Figure 1b shows that it possesses a typical porous, sponge-like structure with a uniform three-dimensional (3D) network morphology. The pore size ranges from 20 to 70 μm, with thin, irregularly shaped pore walls and a relatively smooth surface texture without obvious rough features. This homogeneous structure with high porosity endows pure PVA with excellent water adsorption capacity, making it highly suitable for application scenarios requiring water absorption. However, this feature also brings an obvious drawback: the thin pore walls and loose structure result in low mechanical strength of pure PVA, which limits its application in fields requiring high mechanical robustness.

In contrast, the SEM image of PVA/ANF-2% shows a distinct structural change (Figure 1c). The introduction of ANF significantly reduces the pore size to the range of 10–40 μm, while the pore walls become thicker and more robust, with a rougher surface texture. The overall structure transforms from the loose porous architecture of pure PVA to a denser and more complex network with a more uniform pore distribution. This optimization of the microstructure remarkably improves the mechanical properties of the material. The incorporation of ANF thickens the pore walls and increases the structural density, leading to a significant enhancement in the tensile strength and durability of PVA/ANF-2%, which compensates for the insufficient mechanical performance of pure PVA. The smaller pore size and more uniform pore distribution reduce the risk of structural deformation under external force or environmental changes, enabling higher stability during long-term service [21]. As shown in Figure 1d, the pure PVA sample (PVA/ANF_0_) exhibits a distinct characteristic absorption valley at 3000 cm^−1^, corresponding to the stretching vibration of hydroxyl (-OH) functional groups in PVA. After the addition of ANF, the -OH stretching vibration peak shows a regular shift: the peak of the PVA/ANF 0.1% sample moves to 3190 cm^−1^; as the ANF concentration increases from 0.1% to 1%, the peak position gradually shifts to higher wavenumbers, reaching 3220 cm^−1^ for PVA/ANF 0.5% and 3240 cm^−1^ for PVA/ANF 1%, respectively; when the ANF concentration further increases to 2%, the -OH stretching vibration peak shifts back to a lower wavenumber of 3200 cm^−1^. This trend indicates that at low ANF contents (≤1%), the dispersion of ANF mainly disrupts the self-hydrogen bonding network between PVA molecules, exposing more free hydroxyl groups and leading to an increase in the -OH stretching vibration frequency (higher wavenumber). When the ANF content reaches 2%, a large number of intermolecular hydrogen bonds (O-H···C=O) are formed between the hydroxyl groups of PVA and the carbonyl groups (C=O) on the ANF surface. The enhanced hydrogen bonding reduces the force constant of the O-H bond and lowers its vibration frequency (lower wavenumber), which is in good agreement with the experimental result that the composite organohydrogel achieves the optimal mechanical strength and toughness at 2% ANF content [22,23].

The PVA/ANF composite organohydrogel exhibits excellent mechanical properties due to the incorporation of ANF. To eliminate the interference from factors such as solvent exchange and water content, all samples were immersed in a DMSO/H_2_O mixed solvent with a DMSO mass fraction of 25%, and the water content of the samples was maintained at approximately 80%. In our previous work, PVA hydrogels were prepared by chemical crosslinking and physical crosslinking (freeze–thaw method), respectively, but most of these hydrogels exhibited poor mechanical properties, with limited deformation resistance, load-bearing capacity, and long-term service stability. Therefore, ANF was introduced in this work, which can act as a robust three-dimensional framework to achieve reinforcement and toughening through non-covalent interactions with PVA [24]. The incorporation of ANF not only improves the strength and modulus of the organohydrogel but also increases its elongation at break. As the ANF content increases from 0% to 5%, the elongation at break of the PVA/ANF organohydrogels shows a trend of first increasing and then decreasing. When the ANF content is 2%, the elongation at break reaches the maximum value of 570%, which is 1.66 times that of the pure PVA organohydrogel (344%). This is attributed to the energy dissipation mechanism introduced by the hydrogen bonding between ANF and PVA [25,26]. The integrated area under the tensile strength-elongation at break curve reflects the toughness of the sample. Compared with most hydrogels with low toughness, the ANF-doped composite organohydrogel exhibits excellent toughness. When the mass fraction of ANF reaches 2%, the toughness of the PVA/ANF hydrogel reaches the maximum value of 5.83 MJ·m^−3^, enabling the composite organohydrogel to withstand a wide range of deformation [27,28,29,30]. As shown in Figure 1e,f, the PVA/ANF/NaCl composite organohydrogel achieves the optimal comprehensive mechanical properties at a NaCl concentration of 0.5 M, with a fracture strain of 451.49%, a maximum stress of 2499.5 kPa, a Young’s modulus of 530 kPa, and a fracture toughness of 5.32 MJ·m^−3^, respectively. Therefore, the organohydrogel with 0.5 M NaCl concentration was selected as the optimal sample for all subsequent experiments. The dissolution of NaCl increases the water content in the hydrogel, which may lead to a certain degree of relaxation of the internal molecular structure of the gel, thereby reducing its mechanical strength [31,32]. In addition, after the introduction of NaCl, the hydrogen bonding between PVA and the NaCl solution, together with the inherent ion shielding effect of NaCl, may weaken the interaction between PVA and ANF. Especially at high NaCl concentrations, excessive dissolution of NaCl may destroy the synergistic reinforcing effect between PVA and ANF, resulting in a certain degree of decline in the overall mechanical properties [33]. Nevertheless, the introduction of NaCl brings a significant improvement in electrical conductivity (Figure 1g). By adjusting the concentration of NaCl, the trade-off between mechanical properties and electrical conductivity can be balanced to a certain extent [34,35,36]. Based on the optimized composition (2% ANF and 0.5 M NaCl) which achieves the best balance between mechanical robustness and ionic conductivity, the PVA/ANF/NaCl organohydrogel was fabricated into a flexible strain sensor and a TENG device for various wearable applications.

### 2.2. Distance-Sensing and Pressure-Sensing Properties of PVA/ANF/NaCl Composite Organohydrogels

#### 2.2.1. Distance-Sensing Performance of PVA/ANF/NaCl Composite Organohydrogels

As a composite material with high electrical conductivity, the PVA/ANF/NaCl composite organohydrogel exhibits excellent distance-sensing performance, which is mainly determined by the structure and stability of the conductive network within the gel. Within a small strain range, the PVA/ANF/NaCl composite organohydrogel can detect tiny physical deformations and alter its electrical conductivity accordingly, thereby enabling high-sensitivity distance detection. Specifically, when external pressure or tensile strain is applied, the relative positions of the PVA molecular chains and ANF change, which in turn leads to alterations in the conductive pathways. In the presence of NaCl, ions move freely within the gel, further enhancing its electrical conductivity. With increasing NaCl concentration, the electrical conductivity of the gel is markedly enhanced, rendering the conductance variation in the gel under external force more sensitive and thereby boosting its distance-sensing performance [37].

**Figure 2:**
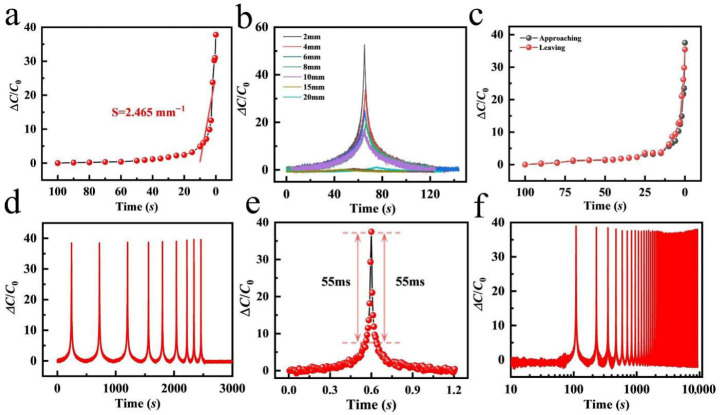
Sensing performance of the PVA/ANF/NaCl composite organohydrogel sensor. (**a**) Schematic diagram of the sensing mechanism of the distance sensor. (**b**) Distance-dependent capacitance response at different distances between the sensor and the aluminum block. (**c**) Capacitance response during approaching and leaving cycles within 100 mm. (**d**) Capacitance variation with time at different moving speeds. (**e**) Response and recovery time of the distance sensor. (**f**) Stability test of the distance sensor.

The target object was lowered until it stopped 0.1 mm above the upper surface of the sensor and then returned to the original position at the same speed. The capacitance change was up to 38 times that of the initial state (Figure 2a). The sensitivity $S_{D}$ is defined as the ratio of the capacitance change rate to the descending distance,

(1) $$S_{D}=\delta (∆R/R_{0})/\delta D$$

where $S_{D}$ is the sensitivity, Δ*R*/*R*_0_ is the resistance change rate, and δD is the vertical distance between the object and the sensor. The sensitivity can reach up to 2.465 mm^−1^ at close range (within 0–10 mm from the sensor). The approaching and moving away processes were plotted as two separate parts to facilitate the analysis of hysteresis and reversibility. As shown in Figure 2b, the capacitance response curves of the aluminum block during descending and ascending are almost identical. The initial position of the aluminum block was adjusted to different heights, and the capacitance response curves of the sensor were highly stable (Figure 2c), indicating excellent reversibility and minimal hysteresis. This demonstrates that the distance sensor can clearly distinguish response signals at different distances. The capacitance response of the sensor remained relatively stable when the object moved away at different speeds (Figure 2d). Notably, the response and recovery time reached 55 ms at a high moving speed, indicating a fast response (Figure 2e). The sensor remained stable after 800 cycles at the same frequency (Figure 2f).

#### 2.2.2. Pressure-Sensing Performance of PVA/ANF/NaCl Composite Organohydrogels

The PVA/ANF/NaCl composite organohydrogel-based pressure sensor was fabricated by sandwiching the as-prepared organohydrogel between two electrodes. The PVA/ANF/NaCl composite organohydrogel served as the dielectric layer, while copper foils were employed as the two electrodes of the sensor. In the absence of applied pressure, the contact area between the copper foil electrodes and the PVA/ANF/NaCl composite organohydrogel dielectric was small. As pressure was continuously applied to the sensor, the contact area between the electrodes and the dielectric gradually increased and continued to expand even after full contact was achieved between the two components. Meanwhile, the distance between the top and bottom electrodes gradually decreased during this process. With the increase in pressure and the resultant expansion of the contact area between the electrodes and the dielectric, oppositely charged ions from the copper foil electrodes and the PVA/ANF/NaCl composite organohydrogel accumulated at the contact interface, forming a high-capacitance electric double layer (EDL). The capacitance varied with the change in the interfacial area formed by ions and electrons [38,39].

**Figure 3:**
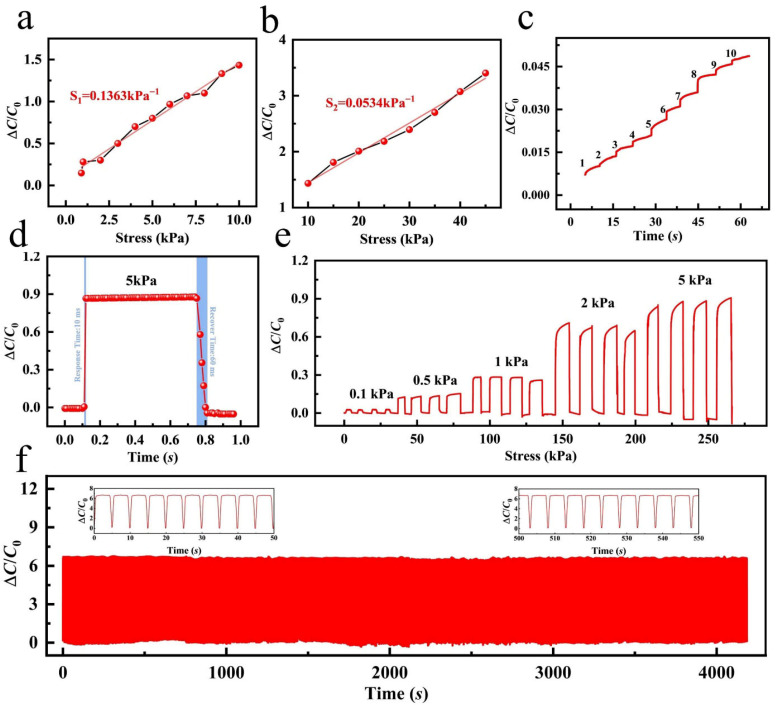
(**a**,**b**) Capacitance change rate under various pressures; (**c**) capacitance change rate corresponding to the tiny deformation induced by simulated raindrops; (**d**) single-cycle response curve under a 5 kPa weight; (**e**) capacitance change rate under different pressures applied by various weights; (**f**) capacitance change rate over 1200 cycles under 5 kPa pressure.

When external pressure is applied, the capacitance rises as the spacing between the two electrodes decreases. Under low pressure conditions, the electrode and dielectric do not achieve full contact. As the applied pressure increases, the contact area between the electrode and dielectric expands, while the electrode spacing is reduced, resulting in a significant increase in capacitance. When pressure reaches a certain level, the electrode and dielectric come into full contact; the contact area no longer increases, and the rate of capacitance change slows down. Sensitivity is defined as the ratio of the relative capacitance change to the applied pressure, and the sensitivity of the pressure sensor increases with rising pressure. The sensitivity of the pressure sensor is generally defined as:

(2) $$S_{P}=\delta (∆C/C_{0})/\delta P$$

where $S_{P}$ is the sensitivity, $∆C/C_{0}$ is the relative capacitance change, and P is the applied pressure. Before full contact between the electrode and dielectric is reached, the sensitivity of the copper foil-based PVA/ANF/NaCl pressure sensor is *S*_1_ = 0.1363 kPa^−1^. Full contact occurs at approximately 2 kPa, beyond which the sensitivity decreases to *S*_2_ = 0.0534 kPa^−1^ (Figure 3a,b). Figure 3c further shows the sensor performance in detecting the weak pressure induced by small water droplets, and the sensor accurately responds to tiny pressure changes, demonstrating a low detection limit and great potential for micro-pressure detection, which enables broad applications in microfluidic detection, environmental monitoring, and other fields requiring high pressure sensitivity. In addition, the response and recovery time of the sensor was evaluated, and under an applied pressure of 5 kPa, the response time is 10 ms and recovery time is 60 ms (Figure 3d), indicating fast response and rapid recovery suitable for dynamic working conditions. The sensor exhibits stable and repeatable responses over a wide pressure range from 0.1 kPa to 5 kPa, with consistent and reliable signals over multiple loading–unloading cycles (Figure 3e) [40]. Long-term stability was verified using 1200 consecutive cycles at 5 kPa, and the capacitive pressure-sensing performance remained stable without obvious degradation (Figure 3f), confirming excellent stability and durability for practical long-term applications [41].

### 2.3. Strain-Sensing Property of PVA/ANF/NaCl Composite Organohydrogels

#### 2.3.1. Strain-Sensing Mechanism of PVA/ANF/NaCl Composite Organohydrogels

The strain-sensing performance of the PVA/ANF/NaCl composite organohydrogel is mainly governed by its resistance variation. Specifically, when external strain is applied to the gel, the internal conductive network of the material undergoes deformation, which in turn alters the conductive pathways and ultimately results in a change in electrical resistance [42,43]. Upon the application of external strain to the surface of the PVA/ANF/NaCl composite organohydrogel, the relative positions between the PVA molecular chains and ANF change, which induces microscopic deformation of the gel’s three-dimensional network structure and further affects the ion transport pathways inside the material. The introduction of NaCl further enhances the ionic conductivity, thus enabling a more pronounced conductance change in the gel under applied strain [44]. The strain-sensing performance of the gel is dominated by the ionic conductive pathways in the PVA/ANF/NaCl composite organohydrogel; as ions migrate through the gel, the electrical resistance of the material varies continuously with the increase in applied strain. Under this sensing mechanism, the strain-resistance response of the gel exhibits favorable linearity, especially in the low strain range, where the resistance change is nearly proportional to the applied strain. With the increase in strain, the conductive network of the material evolves progressively, which significantly boosts the strain response capability of the gel [45].

#### 2.3.2. Strain-Sensing Performance and Applications of PVA/ANF/NaCl Composite Organohydrogels

**Figure 4:**
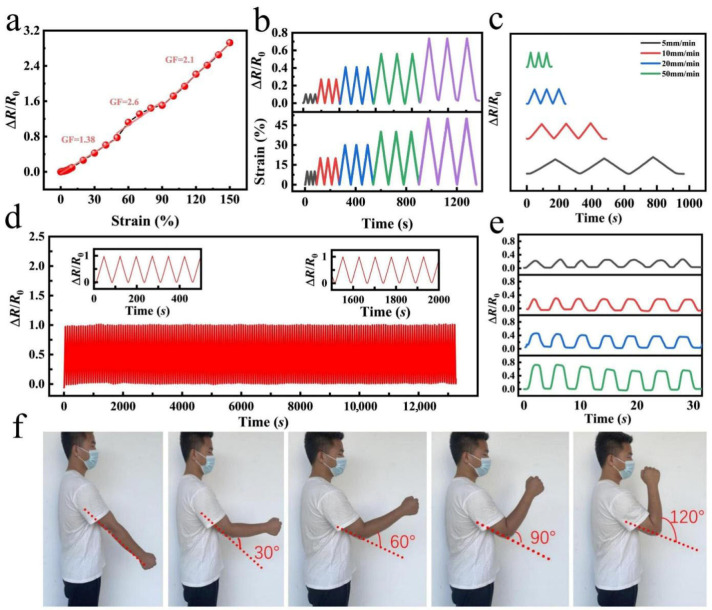
(**a**) Relative resistance changes induced by different applied strains and the corresponding gauge factor (GF) at various strain levels. (**b**) Relative resistance changes corresponding to the applied strain in the range of 0–50%. (**c**) Relative resistance changes under the same pressure at different frequencies. (**d**) Relative resistance changes during 800 stretch-release cyclic strain tests. (**e**) Relative resistance changes at different bending angles with the same movement speed. (**f**) Test process for measuring the relative resistance changes at different bending angles and the relative resistance changes at different movement speeds under the same bending angle.

The incorporation of NaCl significantly enhances the ionic conductivity of the PVA/ANF/NaCl composite organohydrogel, with the most pronounced effect achieved at a NaCl concentration of 0.5 M. The Na^+^ and Cl^−^ ions generated by NaCl dissociation increase the free ion concentration in the gel and boost ion migration, thereby leading to a remarkable improvement in electrical conductivity, and characterization results demonstrate that the gel exhibits a greatly enhanced conductive response to tiny strains at this optimal NaCl concentration. Sensitivity is a core property of the PVA/ANF/NaCl composite organohydrogel for its application as a strain-sensing material, and in this study, the gauge factor (GF) was adopted to quantitatively evaluate its strain-sensing performance, which is defined as the ratio of the relative resistance change ($∆R/R_{0}$) to the applied strain (∆ε) with the calculation formula:

(3) $$GF=(∆R/R_{0})/∆\varepsilon$$

where ∆R is the absolute change in resistance, $R_{0}$ is the initial resistance of the gel without applied strain, and ∆ε is the applied strain. Figure 4a presents the relative resistance changes induced by different applied strains and the corresponding  GF values at each strain level, and the results indicate that the PVA/ANF/NaCl conductive organohydrogel delivers higher sensitivity and stability under high strain conditions, making it suitable for application scenarios requiring large deformation detection; the remarkable increase in GF is closely associated with the NaCl-enhanced ionic conductivity and the structural reinforcement of the gel network by ANF, as the flexible network of the PVA matrix can withstand large tensile deformation under high strain, while the uniform dispersion of ANF further ensures the efficient transmission of deformation signals, enabling the gel to maintain excellent sensing performance over a wide strain range and prominent application potential in large deformation monitoring fields such as large-scale human motion capture and deformation detection of flexible mechanical components, while also exhibiting excellent detection capability for tiny deformations [46,47]. This wide-range sensitivity characteristic originates from the synergistic effect of three core mechanisms: first, the ionic conductivity endowed by NaCl, where free ions provided by NaCl enhance the electrical conductivity of the gel and enable the rapid conversion of mechanical deformation into detectable resistance signals; second, the ANF reinforcement effect, where ANF improves the mechanical strength of the gel and enhances the transmission efficiency of deformation signals via its high aspect ratio and uniform dispersion in the matrix; third, the PVA flexible network, where the elastic three-dimensional structure of the PVA matrix endows the gel with outstanding deformation recovery capability and ensures the stability of sensitivity during repeated cycles. Figure 4b clearly shows the linear relationship between the relative resistance change ($∆R/R_{0}$) and applied strain for the PVA/ANF/NaCl organohydrogel at strains below 50%, and this linear response indicates that the gel has stable electrical properties under small-range deformation, which benefits from the synergistic effect of the NaCl-derived ionic conductive network and ANF-enhanced mechanical stability, enabling the network structure changes induced by deformation to be efficiently converted into linear resistance signal changes. Figure 4c further reveals the effect of tensile rate on the relative resistance change, where the PVA/ANF/NaCl gel shows distinct relative resistance responses at different tensile rates (5 mm/min, 10 mm/min, 20 mm/min, and 50 mm/min), and the relative resistance change presents a non-linear increasing trend with the rise in tensile rate, which is attributed to the influence of deformation rate on the dynamic response of ion migration and network deformation; at lower rates (e.g., 5 mm/min and 10 mm/min), ion migration in the gel network is relatively uniform, leading to a smooth and stable resistance change, while higher rates (e.g., 20 mm/min and 50 mm/min) may cause inhomogeneous local deformation or instantaneous redistribution of ions, which in turn induce fluctuations in the relative resistance change. Figure 4d presents the long-term stability and repeatability of the PVA/ANF/NaCl composite organohydrogel in strain-sensing performance, where after 800 stretch-release cycles at 50% strain, the resistance response of the gel remains highly consistent with no obvious performance degradation observed, indicating that the gel has excellent fatigue resistance and mechanical stability ascribed to the flexible network of the PVA matrix and the reinforcing effect of ANF, and even after long-term cyclic loading, the gel can still accurately respond to external strain changes, exhibiting outstanding durability and reliability [48,49,50,51]. To further evaluate the dynamic response performance of the strain sensor for real-time applications, the response and recovery times at 50% strain were characterized, as shown in Figure S1. The sensor exhibits a response time of ~1 s and a recovery time of ~1 s, demonstrating acceptable dynamic response capability for the low-frequency dynamic applications investigated in this work.

To verify the practical strain-sensing performance of the PVA/ANF/NaCl composite organohydrogel in real-world scenarios, an arm bending test was conducted in this study to systematically characterize its relative resistance change. During the test (Figure 4f), the arm bending angles were set to 0°, 30°, 60°, 90°, and 120°, respectively, the organohydrogel sensor was firmly attached to the lateral side of the elbow to ensure intimate contact with the skin and avoid displacement during movement, and a comfortable short-sleeved T-shirt was worn throughout the test to eliminate interference with the sensor’s performance from clothing. The test results were plotted with time (0–30 s) as the abscissa and relative resistance change as the ordinate to analyze the resistance response capability of the PVA/ANF/NaCl gel at different bending angles, and as shown in Figure 4e, the relative resistance change in the PVA/ANF/NaCl composite organohydrogel increases gradually with the rise in bending angle, while maintaining a stable and gradual response during the whole test process.

### 2.4. Triboelectric Performance and Applications of PVA/ANF/NaCl Composite Organohydrogels

#### 2.4.1. Output Performance of PVA/ANF/NaCl Composite Organohydrogel-Based TENG

A vertical contact-separation mode triboelectric nanogenerator (TENG) based on the PVA/ANF/NaCl composite organohydrogel was fabricated, with aluminum (Al) electrode and polydimethylsiloxane (PDMS) serving as the triboelectric electrodes and the as-prepared organohydrogel as the core functional material [52,53,54]. For output performance characterization, the two triboelectric electrodes were connected in series with an external load resistor. Under open-circuit conditions, no electron transfer takes place between the two electrodes, and the potential difference across the triboelectric pair is linearly proportional to their separation distance. When the electrode separation of the PVA/ANF/NaCl-based TENG increased from 0 mm to 5 mm, the positive open-circuit potential rose rapidly from 0 V to 120 V. The open-circuit voltage was measured using a Keithley 6514 electrometer with an input resistance of 200 TΩ, under which condition electron transfer between the electrodes is negligible. The voltage remained constant before the two triboelectric electrodes approached each other again, while electrons flowed between the two electrodes during the current measurement [55].

**Figure 5:**
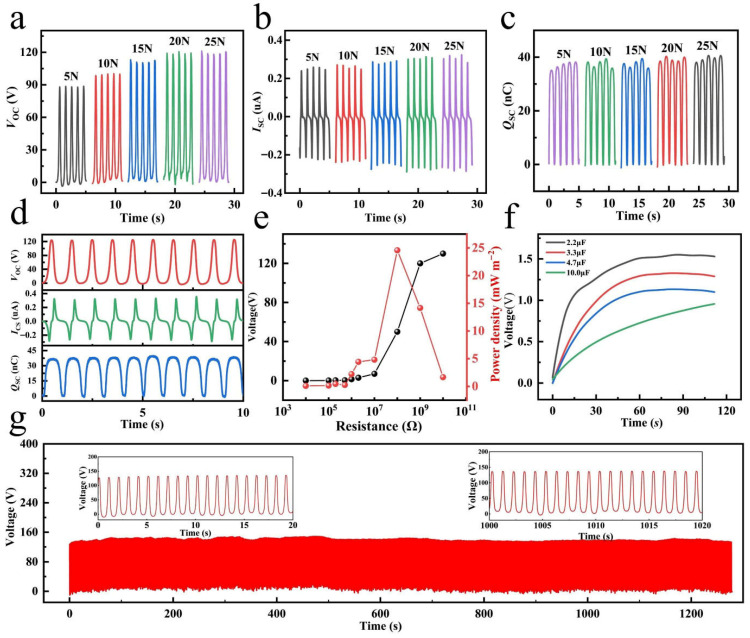
(**a**–**c**) Open-circuit voltage (*V*_OC_), short-circuit current (*I*_SC_), and transferred charge (*Q*_SC_) at 1 Hz under different pressures. (**d**) Output performance of the PVA/ANF/NaCl-based TENG at 25 N and 1 Hz. (**e**) Output power under external loads. (**f**) Output performance under different capacitors. (**g**) Cycling test of the PVA/ANF/NaCl-based TENG over 1100 cycles.

As shown in Figure 5a–c, increasing the applied force from 5 N to 25 N can significantly enhance the effective contact area and compactness between the triboelectric layers, thereby steadily increasing the electrical output. These results fully demonstrate the excellent electrical performance of the PVA/ANF/NaCl-based TENG under optimized frequency and pressure conditions, providing an important basis for its applications in the field of energy harvesting or self-powered sensing [56]. Then, the output performance of the PVA/ANF/NaCl-based TENG was evaluated, and the results are shown in Figure 5d. Based on the excellent electrical and durable properties of the PVA/ANF/NaCl composite organohydrogel, the PVA/ANF/NaCl-based TENG exhibits an open-circuit voltage (*V*_OC_) of 120 V, a short-circuit current (*I*_SC_) of 0.38 μA, and a short-circuit charge (*Q*_SC_) of 36 nC under a periodic external force of 25 N at 1 Hz. As shown in Figure 5e, the PVA/ANF/NaCl-based TENG shows a maximum power density of 23 mW m^−2^ at an external load of 100 MΩ, demonstrating good output performance. In addition, as a renewable energy source for flexible electronic devices, the ability to charge energy storage devices is considered an important indicator for evaluating the output performance of TENGs. Therefore, a bridge rectifier was used to convert the alternating current output by the PVA/ANF/NaCl-based TENG into direct current to charge three capacitors with different capacitances (2.2, 3.3, 4.7, and 10 μF), and the results are shown in Figure 5f. All four charging curves indicate that the PVA/ANF/NaCl-based TENG can maintain stable and continuous output performance within hundreds of seconds (the 2.2 μF and 10 μF capacitors are charged to 1.5 V and 0.9 V within 120 s, respectively). Then, to further verify the output stability of the PVA/ANF/NaCl-based TENG, its output voltage was measured for about 1100 cycles (Figure 5g). The results show that the output performance of the PVA/ANF/NaCl-based TENG is very stable without obvious performance degradation [57].

#### 2.4.2. Applications of PVA/ANF/NaCl Composite Organohydrogel-Based Triboelectric Nanogenerators

**Figure 6:**
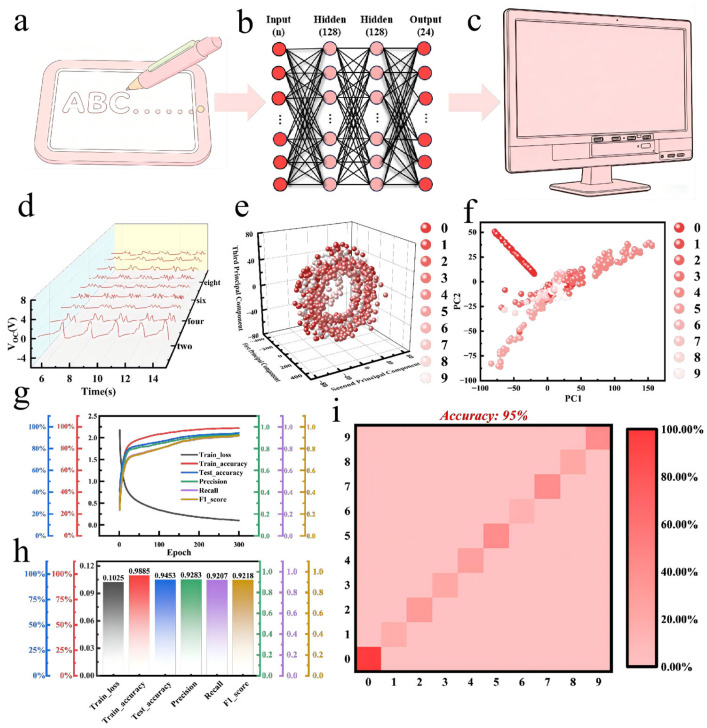
Applications of PAM-based TENG. (**a**–**c**) Schematic diagram of the operating mechanism for an intelligent handwriting pad based on PAM-based TENG. (**d**) Collected TENG writing signals of Arabic numerals 0–9. (**e**,**f**) PCA and dimensionality reduction processing of data. (**g**) Machine learning training process diagram. (**h**) Machine learning training results. (**i**) Smart handwriting pad confusion matrix.

Traditional handwriting recognition methods generally fail to achieve real-time recognition of handwriting signals and suffer from long processing latency. In contrast, triboelectric nanogenerator (TENG) technology can efficiently acquire and identify the dynamic random pressure signals generated during the writing process, which opens up a new avenue for real-time handwriting signal recognition [58,59,60,61]. This study constructs a self-powered smart handwriting recognition system based on the PAM composite organohydrogel TENG (Figure 6a). When localized dynamic pressure is applied to the writing tablet, the time-varying contact-separation process generates a characteristic electrical signal. In the writing scenario, the writing actions of different Arabic numerals induce the TENG to produce signals with unique waveform characteristics by applying dynamic pressure patterns with temporal differences and spatial distributions, as shown in Figure 6d [62]. Based on these collected signals, we constructed a multi-layer perceptron (MLP) neural network classification model with two hidden layers containing 128 neurons each for real-time pattern recognition (Figure 6b,c). To further optimize data visualization and facilitate a clearer understanding of the clustering distribution, principal component analysis (PCA) and dimensionality reduction processing were carried out. The processed data show obvious clustering structures with slight overlaps, as presented in Figure 6e,f. A systematic experimental dataset was established for the 10 Arabic numerals from “0” to “9”. Within a supervised learning framework, after 300 training epochs, the MLP model exhibits excellent performance: the loss function converges rapidly to near zero, and the training accuracy is significantly improved (Figure 6g). Finally, prediction evaluation was performed on the test set, achieving an outstanding test accuracy of 97.6% with high precision, recall, and F1 scores (Figure 6h). The corresponding learning curve and the confusion matrix with an overall accuracy of 97% are displayed in Figure 6g and Figure 6i, respectively.

## 3. Conclusions

This work focuses on the fabrication process, structural characterization, and applications of PVA/ANF/NaCl composite organohydrogels in the field of flexible sensors, with special attention paid to their comprehensive performance in strain sensing, pressure sensing, and triboelectric energy conversion. In this study, a fabrication method combining freeze–thaw crosslinking and physical crosslinking was adopted, and the gel system was optimized and regulated by introducing ANF and NaCl. The experimental results show that the incorporation of ANF significantly improves the mechanical strength of the gel and constructs a uniform and dense polymer network, while the addition of NaCl not only enhances the electrical conductivity of the gel but also endows it with a more sensitive sensing response. Specifically, in the strain sensing performance test, an optimal balance between the electrical conductivity and mechanical properties of the gel was achieved at a NaCl concentration of 0.5 M, the gauge factor (*GF*) increased significantly with the rise in NaCl concentration, making the gel suitable for wide-range strain detection; although NaCl has a slight negative impact on partial mechanical indexes, the overall performance of the gel still meets the requirements for high-sensitivity sensing. In terms of pressure sensing, the relative resistance change in the gel exhibits a favorable linear relationship with the applied pressure through rational regulation of NaCl concentration, which further improves the sensing accuracy. In addition, the triboelectric nanogenerator (TENG) fabricated based on this composite organohydrogel exhibits excellent electrical output and stability, showing broad application prospects in mechanical energy harvesting and intelligent human–computer interaction. Overall, the systematic research in this work not only verifies the excellent performance of PVA/ANF/NaCl organohydrogels in the fields of flexible sensors and TENGs, but also provides a solid material foundation for novel flexible electronic devices such as intelligent wearable electronics and electronic skin, and lays a theoretical and practical foundation for the in-depth research and development of related technologies in the follow-up work. These achievements fully demonstrate the great application potential of PVA/ANF-based composite organohydrogel materials in the field of flexible electronics and provide new ideas and approaches for future technological innovation.

## 4. Materials and Methods

**Materials:** Polyvinyl alcohol (PVA) was procured from Shanghai Aladdin Biochemical Technology Co., Ltd., Shanghai, Shanghai, China. Kevlar pulp was procured from DuPont China Holding Co., Ltd., Shanghai, Shanghai, China. Sodium chloride (NaCl) was procured from Sinopharm Chemical Reagent Co., Ltd., Shanghai, Shanghai, China. Dimethyl sulfoxide (DMSO) was procured from Shanghai Titan Scientific Co., Ltd., Shanghai, Shanghai, China. Potassium hydroxide (KOH) was procured from Sinopharm Chemical Reagent Co., Ltd., Shanghai, Shanghai, China. Deionized water was obtained from a laboratory deionization system.

**Characterizations:** The morphologies and microstructures of the composite organohydrogels were characterized using a scanning electron microscope (SEM, Hitachi SU8010, Hitachi, Tokyo, Japan). The molecular compositions and chemical interactions were identified using a Fourier transform infrared spectrometer (FTIR, Nicolet iS50, Thermo Fisher Scientific, Waltham, MA, USA). The mechanical properties were evaluated using an electronic universal testing machine (Instron 5969, Instron, Norwood, MA, USA). The rheological behaviors were recorded using a rheometer (HAAKE RS150L, Thermo Fisher Scientific, Karlsruhe, Germany). Electrical conductivity was measured using a Four-probe Tester (HPS2526, Helpass Electronic Technologies, Inc., Shenzhen, Guangdong, China). The electrical signals of the pressure sensor and proximity sensor were captured by a digital bridge (TH28320, Tonghui Electronic Co., Ltd., Changzhou, Jiangsu, China). The triboelectric output performance and mechanical energy harvesting performance of the TENG were measured with an electrostatic meter (6517B, Tektronix (Keithley), Beaverton, OR, USA).

**Fabrication of PVA/ANF composite organohydrogels:** As shown in Figure 1, 10 g of PVA powder and 90 g of DMSO were used. The PVA powder was added to DMSO in 5 batches (2 g each time). The above mixture was dissolved under water bath heating at 90 °C with magnetic stirring at 1200 rpm to ensure sufficient dissolution of PVA. The DMSO dispersion of ANF was prepared via proton donor-assisted deprotonation: 2 g of PPTA fibers, 3 g of KOH, 100 mL of DMSO, and 4 mL of deionized water were mixed and mechanically stirred in a sealed environment at room temperature for 4 h until the solution turned dark black, yielding the DMSO dispersion of ANF. A certain amount of ANF DMSO dispersion was mixed with PVA DMSO dispersion, followed by water bath heating at 80 °C and mechanical stirring at 1200 rpm for 2 h. The viscous pale-yellow liquid formed after stirring is the PVA/ANF mixture, where the mass fraction of ANF is 2%.

**Fabrication of PVA/ANF/NaCl composite organohydrogel:** The uniformly mixed solution was poured into a mold for freeze–thaw treatment (freezing at −40 °C for 10 h and thawing for 1 h), and this cycle was repeated three times. After three freeze–thaw cycles, the PVA/ANF composite organohydrogel was removed from the mold and placed in an exchange solution for 24 h to obtain the toughened PVA/ANF/NaCl composite organohydrogel. The exchange solution is a mixed solution of DMSO and deionized water with a mass ratio of 1:2, and the concentrations of NaCl are 0 mol/mL, 0.1 mol/mL, 0.5 mol/mL, 1 mol/mL, and 2 mol/mL.

## Data Availability

The original contributions presented in this study are included in the article/Supplementary Materials. Further inquiries can be directed to the corresponding authors.

## Supplementary Materials

The following supporting information can be downloaded at: https://www.mdpi.com/article/10.3390/gels12050442/s1, Figure S1: Dynamic response and recovery performance of the PVA/ANF/NaCl composite organohydrogel-based strain sensor at 50% strain.
