# Supplementary material for: Preparation, Properties and Application Research of PVA/ANF/NaCl Composite Organic Hydrogel
Source: Gels. 2026 May 19;12(5):442. doi: 10.3390/gels12050442 (PMC13205285; doi:10.3390/gels12050442)
Supplement: Supplementary file 1 [file gels-12-00442-s001.zip › gels-4319095-supplementary.pdf]

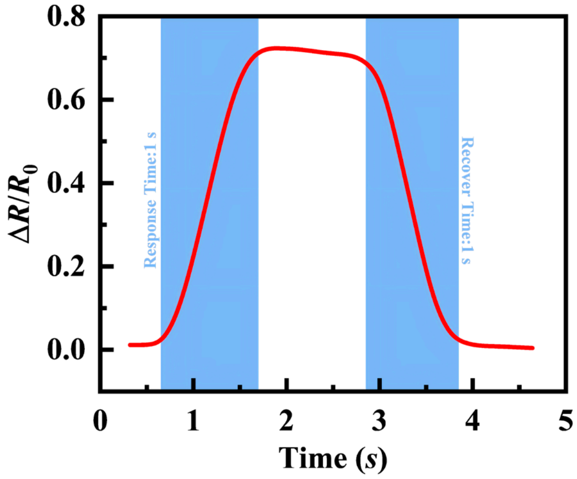

Figure S1. Dynamic response and recovery performance of the PVA/ANF/NaCl composite organohydrogel-based strain sensor at 50% strain
